# M protein from *Streptococcus pyogenes* induces tissue factor expression and pro-coagulant activity in human monocytes

**DOI:** 10.1099/mic.0.2006/003285-0

**Published:** 2007-08

**Authors:** Lisa I. Påhlman, Erik Malmström, Matthias Mörgelin, Heiko Herwald

**Affiliations:** Department of Clinical Sciences, Lund, Section for Clinical and Experimental Infection Medicine, Lund University, SE-22184 Lund, Sweden

## Abstract

Invasive infections caused by the important pathogen *Streptococcus pyogenes* are often associated with disturbed blood coagulation in the human host, and may in severe cases develop into the life-threatening condition disseminated intravascular coagulation. In this study, the addition of M1 protein to human blood or purified peripheral blood mononuclear cells led to a dose-dependent increase of pro-coagulant activity, which was mediated by an upregulation of tissue factor on monocytes. Analysis of the resulting clots by transmission electron microscopy revealed that the cells were covered with a fibrin network that seemed to originate from the cell surface. Taken together, the results imply an important role for M proteins in the induction of haemostatic disorders in invasive streptococcal infectious diseases.

## INTRODUCTION

Sepsis is a severe and often life-threatening condition caused by a systemic activation of the immune system in response to microbial infection. As a result, the infected host can release pathological levels of pro-inflammatory mediators such as cytokines, chemokines and reactive oxygen species, which may lead to haemodynamic rearrangements that ultimately can progress into shock (Riedemann *et al.*, 2003; Van Amersfoort *et al.*, 2003). Notably, septic patients often suffer from varying degrees of local and systemic disturbed haemostasis that can develop into disseminated intravascular coagulation (DIC), a condition characterized by a massive and simultaneous induction of thrombosis and fibrinolysis. It is currently believed that DIC is mainly induced by an activation of the extrinsic pathway, also known as the tissue factor (TF)-driven pathway of coagulation (Levi *et al.*, 2003). Under these conditions, the systemic activation of the extrinsic pathway can lead to the generation of microthrombi that deposit in various organs. In severe cases, thrombus formation will also cause consumption of coagulation factors and platelets, and as a consequence, secondary bleeding may occur that can lead to multiple organ failure and death.

TF is a transmembrane protein that binds to circulating factor VII/VIIa (F VII/VIIa). The TF/F VIIa complex subsequently activates factor X (F X) and factor IX (F IX), leading to thrombin generation, fibrin deposition and platelet activation. TF is found at high levels in adventitial fibroblasts surrounding blood vessels, where it forms a protective barrier against haemorrhage upon vessel injury (Mackman, 2006). While the protein is normally not exposed to human blood, TF can be upregulated on the surface of activated monocytes under pathological conditions (Mackman, 2006), and it has been suggested that in severe infectious diseases, bacteria-induced expression of TF in these cells is a hallmark of DIC (for a review see Doshi & Marmur, 2002). It should be mentioned that various infection models have shown a reduced sepsis-related mortality when animals were treated with tissue factor pathway inhibitor (TFPI) (for a review see Price *et al.*, 2004). In a phase III clinical trial involving patients with severe sepsis, however, application of recombinant TFPI showed no evidence of a survival advantage (Abraham *et al.*, 2003).

*Streptococcus pyogenes* is an important Gram-positive human pathogen that causes a wide array of diseases, ranging from mild infections such as pharyngitis and superficial skin infections, to life-threatening conditions such as necrotizing fasciitis and sepsis (Cunningham, 2000). In order to cause disease, the bacteria express a number of virulence factors, including M proteins, which were as early as in 1969 described to render the bacterium resistant to phagocytosis by immune cells (Lancefield, 1969). M proteins form *α*-helical coiled-coil dimers with a conserved C-terminal end and a highly variable N-terminal end, based on which the streptococcal serotype is defined (for a review see Fischetti, 1989). Today, more than 80 M serotypes have been identified, with the M1 and M3 type being the most common isolates from patients with invasive and toxic streptococcal diseases (for a review see Cunningham, 2000). M proteins are normally anchored to the cell membrane, but can be released from the bacterial surface by the action of host- or bacteria-derived proteinases (Berge & Björck, 1995; Herwald *et al.*, 2004). Thus, the present investigation was undertaken to study the effect of soluble M1 protein on pro-coagulant activity in human blood. Our results show that soluble M1 protein has a pronounced ability to trigger TF upregulation on the surface of human monocytes, which results in the induction of pro-coagulant activity in these cells.

## METHODS

### Reagents.

M1 protein was purified from the growth medium of the isogenic mutant strain MC25, derived from the AP1 *S. pyogenes* strain 40/58 (from the World Health Organization Collaborating Centre for References and Research on Streptococci, Institute of Hygiene and Epidemiology, Prague, Czech Republic). MC25 expresses a truncated M1 protein that lacks the membrane-spanning region, and the protein is therefore secreted into the growth medium (Collin & Olsén, 2000). The purity of the M1 protein preparation was confirmed by SDS-PAGE followed by Coomassie and silver staining. The concentrations of peptidoglycan (PG) and lipoteichoic acid (LTA) in the stock solution of M1 protein (0.5 μg ml^−1^) were below detection limit (<100 ng PG ml^−1^ and <3 ng LTA ml^−1^) as determined by mass spectrometry and ELISA, respectively (Påhlman *et al.*, 2006). M3 protein, M5 protein, M49 protein, protein H, peptostreptococcal albumin-binding protein (PAB), protein L and protein M1 fragments A-S and S-C3 were purified as previously described (de Chateau & Björck, 1994; Kastern *et al.*, 1992; Weineisen *et al.*, 2004; Åkesson *et al.*, 1994). Protein G was obtained from Amersham Biosciences, protein A and LPS from Sigma-Aldrich.

### Purification of peripheral blood mononuclear cells (PBMCs).

PBMCs were isolated from fresh human heparinized blood from healthy volunteers. Blood was diluted 1 : 1 in PBS (138 mM NaCl, 8.1 mM Na_2_HPO_4_, 2.7 mM KCl, 1.5 mM KH_2_PO_4_, 0.5 mM MgCl_2_, 0.9 mM CaCl_2_; Gibco), layered on top of Ficoll-Paque Plus (Amersham Biosciences), and centrifuged at 1000 ***g*** for 20 min at room temperature. The PBMC cell layer was collected and cells were washed twice in PBS.

### Clotting assays.

Human heparinized blood (250 μl) was treated with different bacterial compounds at 1 μg ml^−1^, various concentrations of M1 protein, or medium alone. After an overnight incubation on rotation at 37 °C, cells were washed twice in 135 mM NaCl, 12.9 mM sodium citrate, pH 7.4, in order to remove the plasma. Next, 100 μl of fresh and untreated human citrated plasma was reconstituted with 100 μl 30 mM CaCl_2_. The reconstituted plasma was pre-warmed for 60 s at 37 °C prior to the addition of 100 μl of the washed cell suspension, and the time to form a clot was determined in a coagulometer. Alternatively, 250 μl of PBMCs (2.5×10^6^ ml^−1^ in PBS) was incubated with various concentrations (0.3–20 μg ml^−1^) of M1 protein, fragments A-S or S-C3 (130 nM), or LPS (100 ng ml^−1^; Sigma-Aldrich) overnight at 37 °C, followed by the determination of pro-coagulant activity in normal or F VII-deficient plasma (Hyphen BioMed) as described above. To analyse the intrinsic pathway of coagulation, 50 μl of normal citrated or F VII-deficient plasma was pre-incubated with 50 μl of a kaolin-containing solution (Technoclone) for 60 s at 37 °C. Clotting was initiated by adding 50 μl of a 30 mM CaCl_2_ solution, and the time to form a clot was measured.

### Flow cytometry.

PBMCs (250 μl, 2.5×10^6^ ml^−1^) in RPMI 1640 medium (Gibco) were treated with M1 protein (1 μg ml^−1^ final concentration), LPS (100 ng ml^−1^ final concentration) or medium alone at 37 °C overnight. Cells were then washed in PBS including 2 % (w/v) BSA, and incubated with mouse IgG (Sigma-Aldrich) for 30 min on ice to block unspecific binding of IgG. After two washing steps in PBS with 2 % (w/v) BSA, cells were incubated with FITC–anti-TF IgG (American Diagnostica), a FITC-conjugated isotype control antibody (BD Biosciences), or R-phycoerythrin–anti-CD14 IgG (DAKO) for 30 min on ice. Samples were washed and analysed in a FACSCalibur flow cytometer (Becton Dickinson). Monocytes were identified by side scatter/forward scatter characteristics and CD14 expression (Loken *et al.*, 1990).

### Thin-sectioning and transmission electron microscopy.

Purified PBMCs (5×10^6^ cells ml^−1^ in PBS) were incubated in the presence or absence of M1 protein (1 μg ml^−1^) or fragment S-C3 (0.34 μg ml^−1^) for 20 h at 37 °C. Afterwards, samples were mixed with pre-warmed Ca^2+^-reconstituted human plasma, and were allowed to form a clot. Clots were fixed for 1 h at room temperature and then overnight at 4 °C in 2.5 % glutaraldehyde in 0.15 M sodium cacodylate, pH 7.4 (cacodylate buffer). Samples were then washed with cacodylate buffer and post-fixed for 1 h at room temperature in 1 % osmium tetroxide in cacodylate buffer, dehydrated in a graded series of ethanol, and then embedded in Epon 812 (SPI Supplies) using acetone as an intermediate solvent. When experiments were performed in the absence of calcium, cells were incubated with human plasma, centrifuged (12 000 ***g*** for 30 s) and resuspended in cacodylate buffer. Specimens were sectioned with a diamond knife into 50–70 nm-thick ultrathin sections on an LKB ultramicrotome. The ultrathin sections were stained with uranyl acetate and lead citrate. Specimens were observed in a JEOL JEM 1230 electron microscope operated at 80 kV accelerating voltage. Images were recorded with a Gatan Multiscan 791 CCD camera.

## RESULTS

### M1 protein induces pro-coagulant activity in human blood

**Fig. 1. f1:**
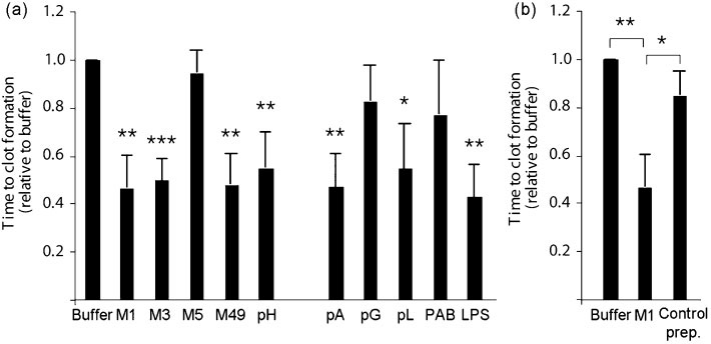
Pro-coagulant activity of human blood in response to bacterial proteins. (a) Human blood was incubated overnight with M proteins of different serotypes (M1, M3, M5 and M49), protein H (pH), protein A (pA), protein G (pG), protein L (pL), PAB or LPS at a final dilution of 1 μg ml^−1^. Samples were then washed, and 100 μl of cell suspension was added to 100 μl of pre-warmed, fresh human citrated plasma, recalcified with 100 μl CaCl_2_. The time to form a clot was subsequently determined in a coagulometer and normalized to buffer-treated cells. (b) Human blood was treated with M1 protein (1 μg ml^−1^), the corresponding concentration of the control preparation, or buffer alone. After an overnight incubation, samples were washed and analysed as above. The values express clotting times relative to the clotting time evoked by buffer-treated cells. Values below 1 indicate increased pro-coagulant activity. Bars represent means and sd from three different donors. Asterisks indicate statistically significant differences as compared to buffer-treated cells, if not otherwise indicated: *, *P*<0.05; **, *P*<0.01; ***, *P*<0.001.

**Fig. 2. f2:**
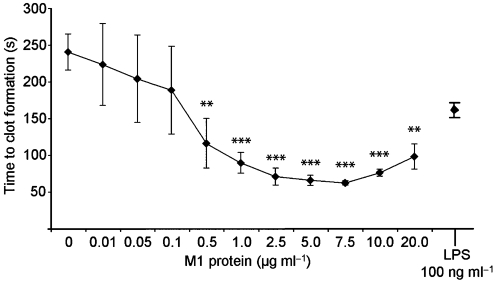
M1 protein induces pro-coagulant activity in human blood. Human whole blood was treated with different concentrations of M1 protein. After 24 h incubation at 37 °C, the samples were washed twice in order to remove M1 protein and plasma components. Then 100 μl fresh, untreated, citrated human plasma was pre-incubated with 100 μl CaCl_2_ at 37 °C for 60 s in a coagulometer. Washed blood cells (100 μl) were added and the time to form a clot was determined. LPS (100 ng ml^−1^) served as a positive control. All samples were analysed in duplicates. The figure shows the mean±sd of three individual experiments. **, *P*<0.01; ***, *P*<0.001.

Disturbed blood coagulation is often seen in patients suffering from severe bacterial infections such as sepsis. In order to evaluate the role of different bacterial proteins in the induction of pro-coagulant activity, human whole blood was treated with surface proteins from various bacterial species and then tested in a clotting assay. After an overnight incubation, blood cells were washed to remove bacterial products and plasma proteins, and supplemented with fresh human plasma. Clotting was then initiated by recalcification, and the time to clot formation was determined in a coagulometer. Fig. 1(a) shows that M proteins from serotypes M1, M3 and M49, but not M5, as well as the M-like protein H from *Streptococcus pyogenes*, induced pro-coagulant activity comparable to the response evoked by LPS. Treatment with protein A from *Staphylococcus aureus* and protein L from *Finegoldia magna* also decreased clotting times in whole blood, whereas protein PAB from *F. magna* and protein G from group C and G streptococci gave no or only weak responses. Based on these findings, and the fact that the M1 serotype is one of the most frequently isolated serotypes from patients with severe and invasive streptococcal infections (Cunningham, 2000), M1 protein was chosen for further studies. It should be noted that the M1 protein that was used was purified from the growth medium of the isogenic M1 mutant strain MC25 that expresses a soluble M1 protein (Collin & Olsén, 2000). To exclude that this preparation was contaminated with other soluble co-purified streptococcal products that may lead to an activation of the coagulation cascade, we subjected the growth medium from an isogenic M1 mutant strain (BMJ71, Kihlberg *et al.*, 1995) that lacks M1 protein and protein H to the same purification protocol. This sample, here referred to as control preparation, was free of M1 protein as confirmed by ELISA, and gave rise to normal clotting times when added to whole blood (Fig. 1b[Fig f1]). The findings imply that the ability to clot human blood was indeed evoked by M1 protein and not by a contaminant. The results also show that, apart from M proteins, other factors derived from Gram-positive bacteria (i.e. protein A and protein L) also have the ability to induce pro-coagulant activity in human blood. However, whether this was induced by the same mechanism as by M proteins was not addressed in the present study. In the next series of experiments, we wished to test whether the effect of M1 protein was dose-dependent. Thus, serial dilutions of M1 protein were added to human blood and the clotting times were determined. Fig. 2 shows that, at a concentration of 0.5 μg ml^−1^, M1 protein already induced a significant pro-coagulant activity in human blood, while a maximal effect was reached at 7.5 μg ml^−1^. Importantly, incubation of M1 protein with human plasma alone did not trigger pro-coagulant activity (data not shown), indicating that blood cells are required in this assay.

### Human monocytes upregulate tissue factor in response to M1 protein

**Fig. 3. f3:**
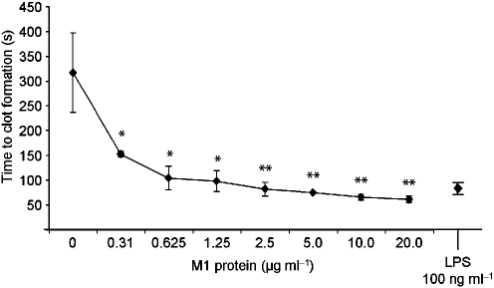
M1 protein induces pro-coagulant activity in human PBMCs. PBMCs were isolated from human blood and incubated with M1 protein at different concentrations or 100 ng LPS ml^−1^ for 24 h at 37 °C. Cell suspensions (100 μl) were then added to 100 μl of pre-warmed citrated human plasma, recalcified with 100 μl CaCl_2_, and the time to form a clot was measured. Values represent the mean±sd of three separate experiments, each done in duplicates. *, *P*<0.05 and **, *P*<0.01.

**Fig. 4. f4:**
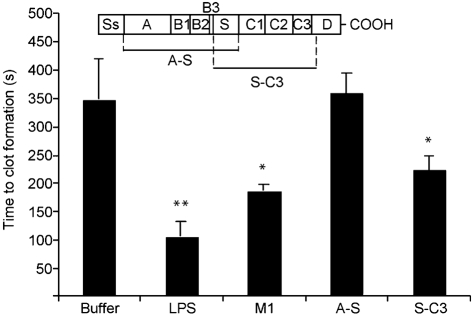
Pro-coagulant activity induced by M1 protein fragments. Human PBMCs (1×10^6^ ml^−1^) were incubated with M1 protein (5 μg ml^−1^) (130 nM), fragments A-S or S-C3 at equimolar concentrations (2.0 and 1.7 μg ml^−1^, respectively), LPS (100 ng ml^−1^) or buffer alone for 20 h at 37 °C, and the ability of the cell suspensions to induce clot formation was thereafter determined in a coagulometer. The figure shows the mean±sd of three individual experiments. Asterisks indicate statistically significant differences relative to buffer control cells: *, *P*<0.05; **, *P*<0.01. Fragments A-S and S-C3 are schematically depicted at the top.

It is now generally believed that blood-borne TF is mainly produced by monocytes (Doshi & Marmur, 2002). Based on our findings with whole blood, we wanted to test whether the interaction between M1 protein and peripheral blood mononuclear cells (PBMCs) is responsible for the induction of pro-coagulant activity in human blood. To that end, human PBMCs were isolated and treated with different concentrations of M1 protein for 20 h. Cells were then added to fresh reconstituted human plasma, and the time to form a clot was determined. As seen in whole blood, treatment with M1 protein caused a significant and dose-dependent acceleration of clot formation (Fig. 3). No clotting was observed when normal plasma was replaced by F VII-deficient plasma (data not shown), implying that the pro-coagulant activity was mediated via the TF-dependent extrinsic pathway of coagulation. To ensure that the intrinsic pathway was still functional, the activated partial thromboplastin time (aPTT) of normal and F VII-deficient plasma was determined, which induced similar clotting times in both plasma samples (data not shown). Moreover, FACS scan analysis confirmed that monocytes upregulate TF in response to M1 protein (data not shown). To localize the domains of M1 protein that are responsible for the induction of pro-coagulant activity, PBMCs were incubated with M1 protein, or fragments A-S or S-C3, and tested in clotting assays. Fig. 4 shows that fragment A-S evoked normal clotting times, whereas fragment S-C3 was almost as potent as the full-length protein. These findings imply that M1 protein induces pro-coagulant activity via the TF-dependent extrinsic pathway, and that the carboxy-terminal region, which is relatively well-conserved among different M serotypes, mediates this effect.

### Monocytes activated by M1 protein are coated by a fibrin network

**Fig. 5. f5:**
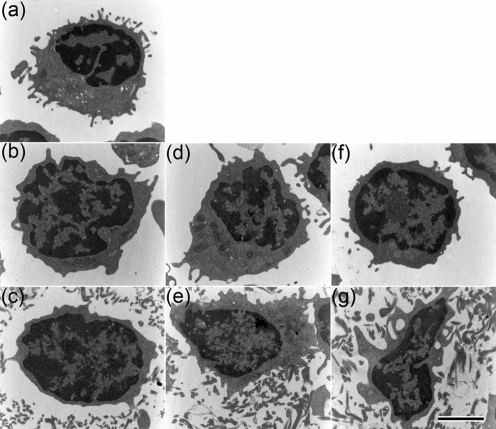
Monocytes treated with M1 protein form a fibrin network on the cell surface. PBMCs untreated (b, c) or stimulated with M1 protein (1 μg ml^−1^) (d, e) or fragment S-C3 (f, g) were incubated with human plasma in the absence (b, d, f) or presence (c, e, g) of Ca^2+^. Pelleted cells (b, d, f) and clots (c, e, g) were fixed, thin-sectioned, and examined by transmission electron microscopy as described in Methods. Monocytes that were not incubated with plasma served as control (a). Bar, 2 μm.

To visualize the effect on clot formation of treating PBMCs with M1 protein, thin sectioning and transmission electron microscopy were employed. PBMCs were incubated with M1 protein or fragment S-C3 overnight, followed by reconstitution with normal plasma in the absence or presence of calcium (Fig. 5d–g[Fig f5]). As controls, PBMCs incubated with normal plasma in the absence of M1 protein or fragment S-C3 (Fig. 5b, c[Fig f5]), and untreated PBMCs (Fig. 5a[Fig f5]), were used. Samples were subsequently fixed, thin sectioned, and analysed by electron microscopy. Fig. 5(e) shows that fibrin polymerization appears to be initiated at the surface of M1 protein-treated monocytes, whereas unstimulated cells were not in contact with the fibrin network (Fig. 5c[Fig f5]). In order to verify that the fibrils consisted of fibrin and not of M1 protein in complex with fibrinogen, cells were stimulated with fragment S-C3, which lacks the fibrinogen-binding site (Åkesson *et al.*, 1994). Similar to M1 protein-treated PBMCs, cells stimulated with fragment S-C3 were in contact with surrounding fibrils (Fig. 5 g[Fig f5]). Moreover, when the experiments were conducted in the absence of calcium in order to prevent fibrin polymerization, no aggregates were found attached to PBMCs (Fig. 5b, d and f[Fig f5]). Taken together, these findings imply that M1 protein can stimulate monocytes to express TF on the cell surface, which in turn serves as an initiation point for fibrin polymerization.

## DISCUSSION

The extrinsic or TF-driven pathway of coagulation is regarded as the most important initiation step of the clotting cascade *in vivo*, and the expression of TF is under normal conditions tightly regulated in order to preserve an intact haemostasis (Mackman, 2006). This situation is altered in severe inflammatory conditions, where pro-inflammatory mediators such as cytokines, C-reactive protein and bacterial endotoxin can induce a systemic upregulation of TF on blood-borne monocytes (Opal & Esmon, 2003). It is now generally believed that these interactions play a key role in the pathogenesis of sepsis-related complications such as the coagulopathy DIC. Moreover, accumulating evidence suggests that TF is also an important modulator of inflammation (Cunningham *et al.*, 1999). For example, mice deficient in TF express significantly lower levels of IL-6 in response to LPS than wild-type animals (Pawlinski *et al.*, 2004). Other studies have shown that active site-inhibited F VIIa (F VIIai) and TFPI reduce the expression of pro-inflammatory cytokines IL-6 and IL-8 *in vivo*, decrease coagulation and thereby prevent death in a lethal baboon model of *Escherichia coli*-induced sepsis (Creasey *et al.*, 1993; Taylor *et al.*, 1998). The pro-inflammatory properties of TF involve protease-activated receptor 2 (PAR-2), which is activated by the TF/F VIIa and TF/F VIIa/F Xa complexes, as well as by F Xa alone (Camerer *et al.*, 2000, 2002). Thus, inflammation and coagulation are intimately connected, and the upregulation of TF under pro-inflammatory conditions is considered as an important mediator of the crosstalk between these two processes.

*S. pyogenes* is one of the most common human pathogens and is responsible for an estimated 616 million cases of pharyngitis and 111 million cases of pyoderma worldwide every year (Carapetis *et al.*, 2005). In addition to these superficial infections, the bacterium is believed to cause 663 000 cases of invasive diseases, resulting in 163 000 deaths, annually (Carapetis *et al.*, 2005). Although rare, invasive streptococcal infections are feared conditions in intensive care medicine due to mortality rates ranging from 25 to 70 % (Carapetis *et al.*, 2005; Stevens, 2003). The pathogenesis of invasive streptococcal infections is often associated with an overamplification of the innate immune system. Notably, even though streptococcal interactions with so-called human effector systems have been studied intensively over the last two decades (for a review see Bisno *et al.*, 2003), the reasons why some, but not all, *S. pyogenes* infections develop into these devastating conditions are still obscure. It has been, however, speculated that gene polymorphisms both in streptococci and the human host could contribute to the severity of the disease. For instance, Beres *et al.* (2006) recently reported that gene polymorphisms, likely to influence M3 protein expression or function, seem to affect the ability of *S. pyogenes* to cause invasive disease, and Kotb *et al.* (2002) reported that variations in MHC class II alleles/haplotypes influence the outcome of invasive streptococcal infectious diseases. A genetic association study performed by Sutherland *et al.* (2005) revealed that a special *tlr2* genotype (16933AA) is associated with significantly increased prevalence of sepsis on admission to the intensive care unit, and specifically increased prevalence of Gram-positive sepsis. Recently, we showed that M1 protein interacts with human monocytes via TLR2 (Toll-like receptor 2) (Påhlman *et al.*, 2006). Since TF expression is under the control of transcription factors such as nuclear factor *κ*B (NF*κ*B) (Guha *et al.*, 2001), which is also activated by TLR2 (Roeder *et al.*, 2004), it is tempting to speculate that *tlr2* polymorphisms may influence the severity of coagulation disorders in invasive streptococcal infections.

Several epidemiological studies have shown that severe infections caused by *S. pyogenes* are associated with certain serotypes, with the M1 and M3 serotypes as the most prevalent (Stevens, 1992). Although an induction of the coagulation system is a common feature of these devastating conditions, little is known about the molecular mechanisms employed by streptococci to impair normal haemostasis. Bryant *et al.* (2003) reported that heat-killed *S. pyogenes* bacteria of serotypes M1 and M3, but not M6, have the ability to stimulate TF and cytokine induction in endothelial cells and monocytes. Our findings that M1 protein alone induces TF-expression in monocytes support these data and show an important role for M proteins in coagulation disorders. Moreover, our data show that *S. pyogenes* bacteria have developed a mechanism that evokes coagulation dysfunction that is not restricted to the site of infection and may result in a systemic activation of the coagulation system. Thus, taken together, our data further emphasize the ability of M protein to induce inflammation and thrombosis, and may help to explain the pathogenesis of sepsis and DIC.

## Abbreviations

- DIC, disseminated intravascular coagulation
- F, factor
- LTA, lipoteichoic acid
- PAB, peptostreptococcal albumin-binding protein
- PBMCs, peripheral blood mononuclear cells
- PG, peptidoglycan
- TF, tissue factor
- TFPI, tissue factor pathway inhibitor
